# Microbe–Mucus Interface in the Pathogenesis of Colorectal Cancer

**DOI:** 10.3390/cancers13040616

**Published:** 2021-02-04

**Authors:** Olivia I. Coleman, Dirk Haller

**Affiliations:** 1 Department of Nutrition and Immunology, School of Life Sciences, Technical University of Munich, 85354 Freising, Germany; dirk.haller@tum.de; 2 ZIEL—Institute for Food & Health, Technical University of Munich, 85354 Freising, Germany

**Keywords:** microbiota, intestinal mucus, microbe–mucus interactions, colorectal cancer

## Abstract

**Simple Summary:**

The human gastrointestinal tract is colonized by a vast number of commensal microbes that are greatly beneficial, but at the same time pose a potential threat to the host. Direct contact of such microbes with intestinal epithelial cells can trigger unfavorable host responses that may, for example, contribute to tumor development. One mechanism of defense against such invaders is the transparent mucus layer that overlies the intestinal epithelium and forms a barrier to separate bacteria from the host. While functioning as a physical barrier, the mucus layer also shapes the composition of the microbial community by providing nutrients and attachment sites. In light of the pivotal role that intestinal microbes and a dysfunctional mucus layer have in gastrointestinal pathologies, including chronic inflammation and colorectal cancer, it is of great importance to understand the intricate mechanisms of microbe–mucus interactions in order to comprehend their contribution to disease pathogenesis and to identify new potential treatment strategies.

**Abstract:**

Overlying gastrointestinal epithelial cells is the transparent mucus layer that separates the lumen from the host. The dynamic mucus layer serves to lubricate the mucosal surface, to protect underlying epithelial cells, and as a transport medium between luminal contents and epithelial cells. Furthermore, it provides a habitat for commensal bacteria and signals to the underlying immune system. Mucins are highly glycosylated proteins, and their glycocode is tissue-specific and closely linked to the resident microbiota. Aberrant mucin expression and glycosylation are linked to chronic inflammation and gastrointestinal cancers, including colorectal cancer (CRC). Aberrant mucus production compromises the mucus layer and allows bacteria to come into close contact with the intestinal epithelium, potentially triggering unfavorable host responses and the subsequent development of tumors. Here, we review our current understanding of the interaction between the intestinal microbiota and mucus in healthy and CRC subjects. Deep knowledge of the intricate mechanisms of microbe–mucus interactions may contribute to the development of novel treatment strategies for CRC, in which a dysfunctional mucus layer is observed.

## 1. Introduction

The human gastrointestinal tract is inhabited by a vast number of microorganisms, termed the intestinal microbiota, that live in a symbiotic relationship with the host. The GI tract represents the most densely colonized organ of the body, with the highest microbial load of 10^11^ bacteria/mL content in the colon [1]. Although many organisms fulfill protective functions and are critical for host physiology, multiple lines of evidence demonstrate that complex shifts in the community structure and abundance of certain microbes are associated with the onset of inflammatory and tumorigenic diseases, such as inflammatory bowel diseases (IBD) and colorectal cancer (CRC) [2,3,4,5]. The body has developed multiple mechanisms of defense to protect itself from the potentially harmful microorganisms residing in the intestinal lumen and to prevent their translocation through the single layer of enterocytes separating the lumen from the host. One such mechanism is the mucus layer overlying the intestinal epithelium, which primarily forms the first line of physical defense, but also functions as a chemical and immunological barrier. A dysfunctional mucus layer is associated with gastrointestinal diseases, including CRC, rendering comprehensive understanding of the complex interplay between the intestinal microbiota and the mucus layer as an essential component towards increasing our mechanistic understanding of disease pathogenesis and thereby opening new avenues for treatment. With CRC being the second most common cause of cancer deaths worldwide [6], novel treatment strategies and alternative approaches are imperative.

## 2. The Intestinal Mucus Layer: Our Knight in Slimy Armor

### 2.1. Mucus Layer Structure and Composition

**Figure 1:**
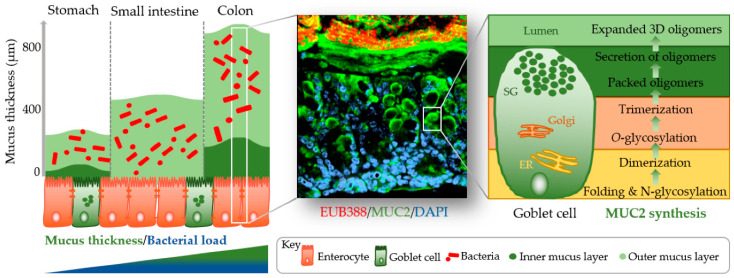
Simplified graphical illustration of mucus thickness and synthesis. Mucus thickness and bacterial load increase from the proximal to the distal end of the gastrointestinal tract. Shown in the left panel are mucus thickness values for the stomach, small intestine, and colon. While the stomach and colon have clearly defined inner and outer mucus layers, the small intestine does not. Shown in the middle panel is a cross-section of a wild-type mouse colon after fluorescent in situ hybridization using a bacterial EUB388 probe (red) and anti-MUC2 staining (green). Nuclei are counterstained with DAPI (blue). Bacteria are clearly confined to the outer mucus layer, with the stratified inner layer being devoid of bacteria. The right panel represents a simplified depiction of MUC2 synthesis in the corresponding goblet cell compartments (ER, endoplasmic reticulum; Golgi, Golgi apparatus; SG, secretory granules).

The identification of methods to visualize and measure mucus allowed for intensive study of the previously overlooked and mostly underappreciated protective layer. Groundbreaking in the understanding of the intestinal mucus layer structure was the development of in vivo mucus thickness measurements in animals [7], which were later followed by ex vivo mucus thickness measurements in human and mouse tissues [8]. Mucus forms a complex viscous secretion that shows distinct structural characteristics along the length of the intestinal tract, reflecting the physiological requirements and the microbial load in the respective intestinal compartments (Figure 1). The oral cavity is covered by a relatively thin (up to 100 µm) salivary film, whereas the stomach mucus layer needs to protect the underlying epithelium from acidic conditions and measures approximately 300 µm in thickness [7,9]. In the small intestine, a relatively thin (100–500 µm), loose, and unattached mucus layer allows for efficient nutrient absorption [7,10]. The colon presents the organ with the thickest mucus layer, measuring around 830 µm, and, in contrast to the small intestine, is composed of an inner stratified layer that is mostly sterile and an outer loose layer that forms a habitat for bacteria [7,11]. This organization is critical for gastrointestinal tract homeostasis, separating most of the luminal microorganisms from the epithelium and the immune system.

Mucus is composed of approximately 95% water, highly glycosylated mucin glycoproteins, lipids, electrolytes, bile salts, antimicrobial enzymes, and immunoglobulins. Mucin proteins form the major building blocks of mucus and are composed of a mucin protein core domain rich in amino acids that form attachments sites for *N*-acetylgalactosamine (GalNAc), which in turn forms extended glycan epitope structures [12,13]. Further to their core domain, mucins can have transmembrane domains that allow cell membrane anchorage [14]. This classifies mucins into cell surface mucins or secreted gel-forming mucins. To date, 21 different mucins have been identified, of which the MUC2-secreted gel-forming mucin represents the major intestinal mucin. Goblet cells of the intestinal epithelium constitutively produce and secrete mucus (Figure 1). In the endoplasmic reticulum (ER) of goblet cells, MUC2 monomers dimerize and subsequently trimerize via C-terminal and *N*-terminal disulfide bridges, respectively [15,16]. These densely packed oligomers are secreted in response to a decrease in Ca^2+^ concentration and increased pH, and subsequently become highly hydrated to form greatly expanded organized sheets that comprise the three-dimensional mucus layer [17]. MUC2 is a highly *O*-glycosylated mucin, with more than 80% of its total molecular weight (2.7 MDa) consisting of oligosaccharide side chains that form a crucial part of microbe–mucus interactions [18,19] discussed in more detail in Section 3. Mucin glycans are mainly composed of *O*-glycosylated (and to a lesser extent *N*-glycosylated) protein cores with glycosyl chains of 2–12 monosaccharides consisting of galactose, fucose, *N*-acetylgalactosamine, *N*-acetylglucosamine, mannose, and sialic acid [20]. Studies in humans and rodents characterizing mucin glycosylation show regiospecificity along the gastrointestinal tract that is relatively conserved between individuals [21].

### 2.2. Mucus Layer Function

As the first line of defense protecting the intestinal epithelium, mucus contributes to the maintenance of epithelial homeostasis and protects against mechanical, chemical, and biological assaults. As a physical barrier, it separates external substances, enzymes, and bacteria from the epithelium. It was recently suggested that intestinal mucus forms three lines of defense against bacteria [22]. Firstly, through the physical inner mucus layer barrier, secondly, via the sentinel goblet cell response, and thirdly, through the crypt goblet cell-emptying response. Particularly in the colon, the inner mucus layer forms a size-exclusion filter that separates the intestinal microbiota from the host [11]. Consequently, intestinal bacteria are kept at a distance from the epithelium due to IgA-induced bacterial aggregates that are too large to diffuse through the colonic mucus layer [23]. In case the first mucus defense barrier is breached, specialized sentinel goblet cells that are situated along the top of intestinal crypts respond by secreting a mucus plume to wash away penetrating bacteria [24]. Following this response, crypt goblet cell emptying is the last attempt to protect the epithelium from the invading bacteria [25]. At the same time as forming a physical hurdle, the mucus layer simultaneously acts as a diffusion barrier that allows ions, nutrients, and water to reach the enterocytes and provides nutrients and attachment sites for the intestinal microbiota [26,27]. This relationship between the microbiota and mucus is very intricate. A recent publication by Bergstrom et al. has added another functional aspect to mucus, demonstrating that proximally derived *O*-glycosylated mucus encapsulates fecal material and the microbiota to modulate microbiota structure and function, as well as transcription in the colon mucosa [28]. Interestingly, the microbiota directs its own encapsulation by inducing *Muc2* production from proximal colon goblet cells [28]. This work has also introduced a major revision to the current mucus system model of locally produced mucus through the identification of two distinct *O*-glycosylated entities of MUC2: a major form produced by the proximal colon that encapsulates and shapes the microbiota and a minor form derived from the distal colon that adheres to the major form [28]. Its high water content renders the mucus layer as a lubricant that protects against dehydration and mechanical stress [29]. Importantly, intestinal mucus also forms the first line of immunological defense limiting exposure to antigens and bacteria and through direct interaction between mucin glycans and immune cells via lectin-like proteins [26,30]. MUC2 was found to imprint dendritic cell tolerance, implying an important role of glycosylated mucin domains in tolerogenic mechanisms [31].

## 3. The Mucin Glycocode: Facilitator of Microbe–Mucus Interactions

The plethora of variations in the precise interplay of glycosyltransferases involved in *O*-glycan synthesis allows for an enormous structural variability in mucin glycans, which present a form of glycocode [32], which may serve as an interspecies communication facilitator between microbes and the host. These mucin glycans represent potential attachments sites and an energy source to intestinal microbes, thereby acting as a facilitator of microbe–mucus interactions. By providing attachment sites and a source of nutrients through the intestinal mucus, the host likely selects its commensal microbiota, rendering intestinal mucus as one of numerous factors (as, for example, antimicrobial peptides and dietary factors) that control species- and site-specific microbial composition at the epithelial interface. In a symbiotic state of homeostasis, this preserves health, while in a dysbiotic milieu, it seems feasible that opportunistic bacteria or pathogens may alter host mucus in such a way that it forces the host to “select” a different microbial community that potentially drives disease pathology.

### 3.1. Mucin Glycans as a Bacterial Attachment Site

The ability to attach to the host is a prerequisite for colonization and prolonged gastrointestinal residency of microbes [33]. Adhesion of commensal bacteria to intestinal mucus benefits the host as it is suggested to be one of the mechanisms for host colonization resistance of pathogens, achieved by competing for attachment sites, producing antimicrobials, modulation of immune responses, reducing oxygen levels, and depleting nutrients [34]. Microbes express adhesins that enable attachment to mucus, including extracellular appendages, such as pili and flagella, as well as specific mucus-binding proteins (MUBs) (reviewed in [35]). The gram-positive bacterium and well-established probiotic strain, *Lactobacillus rhamnosus* GG was shown to express mucus-binding pili on its surface, with pilin subunits shown to either directly bind to mucin domains or bind through electrostatic contacts [36]. Flagella have also been reported to display adhesive properties to mucus in both pathogenic and probiotic strains [37,38]. In another example, *Bifidobacterium infantis* was shown to harbor oligosaccharide-binding proteins, which facilitate the bacterial mucus-binding ability [39]. In gnotobiotic mice colonized with *Bacteroides fragilis* and *Escherichia coli*, *B. fragilis* were found in the mucus layer, while *E. coli* were restricted to the lumen [40]. Further analysis showed that *B. fragilis* specifically binds to highly purified mucins, suggesting mucus binding as a likely mechanism for intestinal colonization [40]. MUBs are extracellular adhesion effector molecules of lactobacilli [41], with the best-studied example being the 353-kDa MUC produced by *Lactobacillus reuteri* ATCC 53,608 that interacts with specific muco-oligosaccharides [42,43]. Their molecular nature and precise function in vivo remain to be elucidated.

### 3.2. Mucin Glycans as a Bacterial Energy Source

The permanently renewing intestinal mucus layer represents an important ecological niche rich in nutrients, providing a particularly beneficial environment to commensal bacteria. The use of host-derived mucin glycans as an energy source becomes particularly important when dietary glycans are sparse. A clear growth advantage in such scenarios is evident for metabolically flexible commensal bacteria that are able to sequentially degrade mucin *O*-glycans for utilization as carbon and energy sources. This degradation is governed by the specific enzymes produced by the commensal bacteria or pathogens, including esterases, glycosidases, sulfatases, and specific mucinases that cleave the protein backbone [44,45]. Bacteria recognize compact mucin glycan structures and degrade the individual glycans to yield short-chain fatty acids (SCFAs) that diffuse through the inner mucus layer and present an energy source for the intestinal epithelial cells. The harvest of degraded glycans for their own metabolism presents a colonization advantage for bacteria. At the same time, this glycan degradation makes oligosaccharides available to non-mucin degrading bacteria as part of a microbial food chain, therefore maintaining the intestinal microbiota as a whole. Mucin degradation was initially associated with pathogenicity [46,47]. Since then, it has become apparent that a large portion of the genome of certain commensal bacteria, including *Bacteroides thetaiotaomicron*, *Barnesiella intestinihominis*, *Ruminococcus gnavus*, and *Akkermansia muciniphila*, is dedicated to complex carbohydrate degradation and utilization [48,49,50,51]. Martens et al. identified that *B. thetaiotaomicron* contains polysaccharide utilization loci (PULs) that are upregulated when grown on *O*-glycans and showed that *B. thetaiotaomicron* mutants for *O*-glycan PULs are outcompeted by wild-type strains in mice fed a simple sugar diet [52]. These findings demonstrate *B. thetaiotaomicron* requires glycans, including mucins, for successful colonization. Ironically, while the *B. thetaiotaomicron* sialidase harvests sialic acid from mucin glycans, the bacterium is unable to utilize sialic acid, making it available to and promoting the growth of other bacteria, including the enteric pathogens *Clostridium difficile* and *Salmonella typhimurium* [53]. Another well-known mucin-degrading specialist is *A. muciniphila*, an abundant resident of the human gut [54,55]. An in vitro study investigating *A. muciniphila*’s colonization preferences and response to environmental parameters, such as pH and mucins, showed that mucins as a nutritional source are a more important modulator of the microbiota composition than pH [56]. Authors observed higher levels of *Akkermansia*, *Bacteroides*, *Ruminococcus*, *Sutterella*, and *Arthrobacter* in a cluster of mucin-rich bacterial communities that was significantly different from that of mucin-deprived communities [56]. It is well-accepted that host factors (including mucus and antimicrobial peptides), diet, age, and the mode of birth represent examples of factors that shape the composition of the intestinal microbiota and its modulation [57]. For example, a systematic review of clinical trials concluded that an increase in abundance of *A. muciniphila* was observed following dietary modulation through caloric restriction, supplementation with pomegranate extract, resveratrol, polydextrose, or sodium butyrate [58], rendering diet as one important modulator of this mucin-degrading specialist. *A. muciniphila* has been shown to possess probiotic properties, prevent or treat metabolic disorders, reduce metabolic inflammation, and restore the gut barrier [59,60,61], contributing to the maintenance of mucosal integrity. A study maintaining mice on a polysaccharide-deficient diet demonstrated that the mucin-degrading generalist *B. thetaiotaomicron* turns to host mucus glycan foraging when polysaccharides are absent [49]. In line with this, Desai et al. were able to show that a diet deficient in complex plant fiber promotes expansion and activity of the mucin-degrading bacteria *A. muciniphila* and *Bacteroides caccae* in a synthetic human gut microbiota assembled in a gnotobiotic mouse model [62]. This shift in mucin-degrading bacteria was shown to alter the status of the colonic mucus barrier and susceptibility to *Citrobacter rodentium*–induced colitis [62]. Findings support a model of triangular interplay between dietary fiber, intestinal microbiota metabolism, and intestinal mucus, which may impair the intestinal mucus barrier and increase susceptibility to pathogens. The presence and activity of mucin-degrading bacteria in the mucus layer may have strong positive and negative effects on host health, highlighting the need to understand the role of mucins in microbial community dynamics and microbe–host interactions.

## 4. The Microbiota as a Modulator of Intestinal Mucus

The interaction of microbes and intestinal mucus is bidirectional, where not only mucus and mucin glycans select the microbiota composition, but where the intestinal microbiota shapes mucus properties. Evidence of a direct effect of the intestinal microbiota on mucus layer properties was demonstrated by the requirement of meprin β, a protease activated upon bacterial exposure, for small intestinal mucus release [63]. Furthermore, the modulation of the mucin glycan profile in the presence of bacteria has also been observed (reviewed in [51]). The density of the intestinal microbiota forms a gradient along the length of the intestinal tract, reaching its highest load of 10^11^ bacteria/mL content in the colon [1]. The observation that both the intestinal mucus thickness and the microbial load increase towards the distal end of the intestinal tract [1,7] provides evidence for a clear association between the two. In line with this, mucus is thinner and penetrable to microbiota-sized beads in germ-free animals, and its secretion can be stimulated through exposure to such bacterial products as lipopolysaccharides (LPS) and peptidoglycans (PGN) [64]. Conserved microbe-associated molecular patterns (MAMPs) can be recognized by intestinal epithelial cells through a family of innate immune system receptors called toll-like receptors (TLRs), most of which signal by recruiting the key adaptor protein myeloid differentiation factor 88 (MyD88) to initiate signaling cascades involved in inflammatory and tissue renewal and repair responses [65].The importance of TLR family members in influencing mucus properties was demonstrated in intestinal epithelial-specific myeloid differentiation primary response 88 knockout mice (*IEC-Myd88^−/−^*), which showed decreased mucus production [66]. A deficiency of MyD88 has been shown to cause increased susceptibility to chemically induced colitis and infectious colitis, with *IEC-Myd88^-/-^* mice displaying exaggerated tissue damage, reduced antimicrobial responses, and impaired goblet cell responses [66,67,68]. Furthermore, MyD88 deficiency in the Apc^Min/+^ mouse model of spontaneous intestinal tumorigenesis demonstrated that MyD88 signaling substantially contributes to tumor growth [69]. A further example demonstrating the importance of receptor signaling on intestinal mucus properties is provided by vitamin D/vitamin D receptor (VDR) signaling. Vitamin D/VDR signaling has increasingly been recognized to play a role in intestinal homeostasis to modulate the intestinal barrier, the microbiota, and immune responses [70]. Evidence suggests a beneficial role of vitamin D/VDR signaling in experimental and clinical IBD, attributed to alterations in the microbiota [71,72,73]. Amongst other functions, vitamin D/VDR signaling regulates antimicrobial peptide levels in intestinal mucus [74,75]. Furthermore, studies have demonstrated a role for vitamin D/vitamin D receptor signaling in modulating mucus secretion through the regulation of Ca_2_+ assimilation [72,76]. Evidently, vitamin D/VDR signaling, the intestinal microbiota, and the intestinal mucus layer are connected through a complex interplay to maintain epithelial homeostasis. A study investigating the modulation of intestinal mucus by commensal bacteria demonstrated that a period of six weeks is required for the colonic inner mucus layer to become impenetrable to bacteria following the colonization of germ-free mice [77]. Furthermore, this study showed that an additional two weeks (eight weeks in total) of colonization are required to reach a bacterial composition of conventionally raised mice. Together, these findings demonstrate the complex dynamics of mucus layer development and conventionalization in germ-free mice, indicating that studies investigating mature microbe–mucus interactions and characteristics of the latter should therefore be performed after a minimum eight-week colonization period. The comparison between two genetically identical mouse colonies housed in separate rooms of the same specific pathogen-free animal facility revealed that the microbiota composition differed between the two locations and affected inner mucus layer penetrability [78]. The transfer of cecal microbiota from these mice to germ-free mice transmitted the microbiota-induced mucus phenotype [78]. These findings demonstrate that the microbiota and its community structure directly affect mucus barrier properties, with potential implications for disease. A dysfunctional mucus layer may allow bacteria to come into direct contact with the epithelium, triggering adverse host responses, such as an inflammatory response, and allowing bacteria that are uncharacteristic for this milieu to find a niche and flourish. What remains to be fully understood is the exact mechanism by which bacteria trigger mucus development and mucus release and which members of the intestinal microbiota form key players in this process.

## 5. The Microbe–Mucus Interface in CRC

Accumulating evidence unarguably renders the intestinal microbiota as an important player in the development and progression of CRC. With mucins presenting the most prominent host molecules involved in microbe–host interactions, intense research efforts to understand microbe–mucus interfaces are warranted, particularly for pathologies such as CRC that display both microbial dysbiosis and aberrant mucus characteristics. While CRC is characterized by a progression from adenoma to carcinoma, its development may also follow the events of IBD, termed colitis-associated colorectal cancer (CAC). This section will summarize our understanding of mucus characteristics associated with IBD, CAC, and CRC and of the bidirectional and potentially detrimental interaction between mucus and the intestinal microbiota in such scenarios (see also summary Table 1).

Numerous studies clearly associate a dysfunctional intestinal mucus layer with gastrointestinal pathologies such as IBD and cancer. Mice defective in the atonal homolog 1 (Atoh1) transcription factor which is required for intestinal secretory (goblet, Paneth, enteroendocrine) cell differentiation display increased intestinal tumor predisposition [79]. This confirms the necessity for mucin-producing goblet cells in the protection against tumorigenesis. The generation of *Muc2* knockout mice (*Muc2*^−/−^) provided direct evidence that a lack of this main mucus layer component results in the development of spontaneous CRC [80] and colitis [81]. Ulcerative colitis (UC) is a form of IBD, in which contact with bacteria and their antigens triggers unfavorable immune responses and drives inflammation [73,82]. In several different murine models of colitis as well as in colon biopsies of UC patients, the intestinal mucus layer was shown to be compromised [83,84]. Mucin glycosylation is altered in active UC, with a shift towards smaller and less complex glycans, but returns to normal glycosylation patterns as inflammation fades in inactive UC [85]. Authors also found that the extent of glycan repertoire alterations positively correlated with disease severity [85]. In line with this, the mucus layer is more penetrable to bacteria in murine colitis models and UC patients [82]. Interestingly, mucus layer penetration in UC patients in remission was comparable to healthy controls [82]. Bergstrom et al. demonstrated that intestinal mucin *O*-glycosylation is essential to prevent bacterial intrusion and caspase-1 inflammasome activation, thereby protecting against colitis and CRC [86]. As mucin glycosylation in the human colon is stable over time, it forms an important factor for the selection of the intestinal microbiota. It seems feasible that dysbiosis of the intestinal microbiota is linked to the altered mucin glycan profile observed in UC. However, the lack of these mucus changes in Crohn’s disease (CD) which forms the other major form of IBD, where an increase in goblet cells and a thicker mucus layer are observed [87], demonstrates that these changes are not a universal consequence to inflammation and that the causal relationship between inflammation and intestinal mucus abundance remains poorly understood.

Similarly to the IBD phenotype, both CAC and CRC display impaired mucus characteristics, such as altered mucin expression [88,89,90] and atypical glycosylation [91,92]. The inverse correlation of the mucin-degrading specialist *Akkermansia* with gastrointestinal diseases render it a potential biomarker for a healthy intestine [50]. Recent studies have shown an increase in *A. muciniphila* in CRC patients, which may be a consequence of the overexpression of certain types of mucins in CRC [93,94]. Interestingly, mucus plays a dual role in CRC, whereby its protective nature prevents CRC development, but at late stages of malignancy, it contributes to tumor growth and disease progression [95]. Of note here is the existence of mucinous colorectal cancer (MCC), which is observed in a subset (10–15%) of CRCs, associated with a proximal location, and characterized by more than 50% extracellular mucin [96,97]. Normal intestinal epithelial cells display apical expression of mucins. In cancer cells, mucus surrounds the cell surface to assist in immune evasion, facilitate attachment and invasion, and reduce anti-cancer drug efficacy [98]. A disrupted mucus barrier allows increased mucus layer penetration of bacteria, which may form bacterial biofilms in which different bacteria, often uncharacteristic for the niche, cooperate to ensure successful establishment. Such intestinal biofilms present an important factor contributing to CRC [99,100,101]. For example two carcinogenic subtypes *pks+ E. coli* (encodes the genes responsible for colibactin genotoxin) and enterotoxigenic *Bacteroides fragilis* (ETBF, encodes genes for the *Bacteroides fragilis* toxin) were shown to cooperate together to induce tumors in co-colonized mice, compared to mono-colonized mice [100]. The reduction of the mucus layer by ETBF allowed *pks+ E. coli* to come into close proximity with the intestinal epithelium [100]. These findings suggest that a co-expression analysis of the *Bacteroides fragilis* toxin and colibactin may have value in general screening and potential prevention of CRC.

The increased mucus layer penetration of bacteria as observed in inflammation and cancer causes a breach and presents a trigger of the three lines of coordinated mucus defense strategies described in Section 2.2, which quickly lead to mucus exhaustion. This presents the host with the demanding and time-consuming task of regenerating large MUC2 glycoproteins, inevitably leading to ER stress. Two goblet cell mutant mouse lines with mutations in the *Muc2* gene (Winnie and Eeyore) develop mild spontaneous colonic inflammation and chronic diarrhea [102]. In these mice, an incompletely assembled MUC2 precursor accumulates in the ER of goblet cells, inducing ER stress. In line with this, the authors showed that UC patients displayed a similar accumulation of the MUC2 precursor, with ER stress observed even in non-inflamed UC tissue of the same patients [102]. Aberrant mucus production that likely results from ER stress subsequently diminishes the mucus layer barrier to trigger inflammation. The unfolded protein response regulator X-box-binding protein 1 (XBP1) is associated with IBD, and it was shown that *Xbp1* knockout mice display goblet cell deficiency and aberrant mucin secretion [103]. In these mice, *Xbp1* deletion causes spontaneous enteritis and increased susceptibility to induced colitis secondary to defects in Paneth cells and goblet cells [103]. In another study, it was shown that the ER-localized protein disulfide isomerase anterior gradient 2 (AGR2) is required for the maturation and secretion of MUC2 in murine colonic goblet cells, with mice lacking AGR2 showing an increased rate of rectal prolapse and higher susceptibility to chemically induced colitis. [104]. It seems that goblet cell maturation and function can be a target of ER stress in the context of inflammation as well as being a direct participant in the development of inflammation as a consequence of ER stress. In our own transgenic murine model of activating transcription factor 6 (ATF6)-induced microbiota-dependent CRC (nATF6^IEC^), we showed a loss of mucin-filled goblet cells and a more penetrable mucus layer already preceding tumor formation in the colon of homozygous nATF6^IEC^ mice [105]. Surprisingly, in this mouse model, the observed mucin depletion and microbial penetration were not associated with an inflammatory response. Inflammation was only observed at late tumor stages. Furthermore, germ-free housing demonstrated that the aberrant mucus phenotype requires bacteria as an additional trigger to ER stress [105]. Findings may indicate that the observed bacterial penetration likely results from a combination of increased bacterial glycan degradation and subsequent ER stress-induced host defects in mucus production rather than being a sole consequence of the latter. Here, nATF6 signaling represents an intrinsic mechanism of tumorigenesis, with exogenous mechanisms triggered through interactions of bacteria with intestinal epithelial cells. The presence of bacteria, particularly of aggressive bacteria, and bacterial products may result in the secretion of sub-optimally assembled mucus due to an increased demand of mucus and ER stress, generating a more penetrable inner mucus layer. In support of this, the transfer of cecal content from nATF6^IEC^ tumor mice into germ-free nATF6^IEC^ mice lead to higher tumor incidence compared to cecal content from healthy controls [105]. Similarly, and as summarized in Section 4, the colonization of germ-free mice with cecal content from mice that displayed a partially penetrable mucus layer transferred the mucus phenotype to recipient mice [78].

**Table 1:** Summary of mucus characteristics and microbe–mucus interactions observed in IBD and CAC/CRC.

Pathology	Mucus Characteristics	Experimental Evidence
**IBD**	Compromised structure and increased bacterial penetration of mucus layer	*Muc2*^−/−^ mice develop spontaneous colitis [81].
		Murine colitis models and UC patients show increased bacterial penetration [83,84].
	Altered mucin glycosylation	Smaller, less complex glycans in active UC [85].
	Aberrant mucin secretion	Intestinal *Xbp1* knockout mice display spontaneous enteritis, increased susceptibility to colitis, goblet cell deficiency, and aberrant mucin secretion [103]. See also [83,84].
	Accumulation of the MUC2 precursor	Mutant *Muc2* gene (Winnie and Eeyore) mice develop inflammation and MUC2 precursor accumulation. UC patients (inflamed and non-inflamed) accumulate the MUC2 precursor [102].
**CAC/CRC**	Aberrant mucus layer and increased mucus layer penetration	*Muc2*^−/−^ mice develop spontaneous CRC [80].
		Increased tumor predisposition in mice defective in the secretory cell lineage differentiation transcription factor atonal homolog 1 (Atoh1) [79].
		nATF6^IEC^ mice develop spontaneous colonic tumors and show a microbiota-dependent mucin-filled goblet cell loss and increased bacterial penetration [105].
	Atypical glycosylation	Altered mucin *O*-glycan structures including, for example, changes in core glycan structures and Tn antigens [91,92].
	Altered mucin expression and atypical extracellular mucin expression	Abnormal subcellular distribution, de novo expression, and overexpression of mucins [88,89,90].
		Mucus completely surrounds cancer cells [96,97,98].
	Bacterial biofilm formation and bacteria-induced mucus alteration	Bacterial biofilms contribute to CRC, e.g., carcinogenic subtypes *pks+ E. coli* and enterotoxigenic *Bacteroides fragilis* cooperatively induce tumors in co-colonized mice. The reduction of the mucus layer by ETBF allowed *pks+ E. coli* to come into close proximity with the intestinal epithelium [99,100,101].
		Increase in the mucin-degrading specialist *A. muciniphila* [93,94].

## 6. Experimental Models to Investigate Microbe–Mucus Interactions in the Intestine

**Figure 2:**
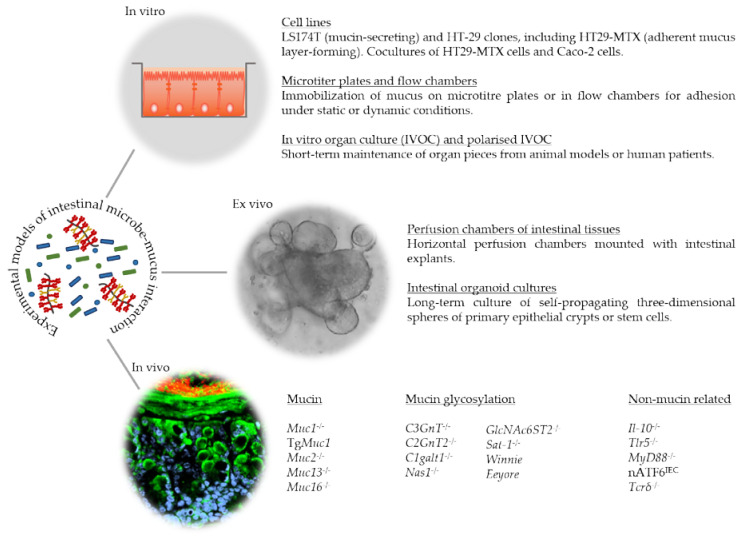
Examples of experimental models of intestinal microbe–mucus interactions. Mucus-secreting and mucus layer-forming cell lines, in vitro organ culture (IVOC) and polarized IVOC, as well as microtiter plates and flow chambers serve as useful models to apply in vitro. The long-term culture of intestinal organoids and short-term culture of intestinal explants in perfusion chambers present two ex vivo models. Listed are mucin, mucin glycosylation, and non-mucin-related rodent models that display alterations in the mucus layer, providing useful in vivo tools.

What becomes evident from the studies summarized in this review is the need for models for the successful study of intestinal microbe–mucus interactions. To this end, a range of in vitro and ex vivo assays, cell lines, and organ cultures, as well as in vivo rodent and non-rodent models are available (recently reviewed in [106]). Examples of these models are summarized in Figure 2.

The colon carcinoma cell line LS174T secretes MUC2 (major colon mucin) and MUC5AC (major stomach and gallbladder mucin) [107]. Numerous studies have used this cell line to study specific host–bacteria interactions and effects on mucus characteristics [108,109,110]. The added advantage of the adenocarcinoma cell line HT29-MTX, the second cell line commonly used to study microbe–mucus interactions, is the formation of a mucus layer overlaying the epithelial cells that are composed of mature goblet cells [111]. Studies using this cell line have identified particular bacteria and their bacterial products that alter mucus production and glycosylation including, but not limited to, [112,113,114,115,116]. For example, Mack et al. used coincubation experiments to show that probiotic *Lactobacillus* strains adhere to HT29-MTX cells and upregulate *MUC3* transcription and secretion to diminish enteropathogenic *E. coli* (EPEC) adhesion [117]. The coculture of HT29-MTX cells and the enterocyte-like Caco-2 cell line more closely resembles the intestinal epithelium and produces a similarly continuous mucus layer [118]. One study demonstrated that the presence of the mucus layer in Caco-2/HT29-MTX cocultures decreased the adhesion of *L. rhamnosus* GG, *Bifidobacterium*, *E.coli,* and *Listeria monocytogenes* compared to Caco-2 cell-only cultures [119]. Binding of bacteria to mucus and mucins can be efficiently investigated under static conditions using microtiter plates and under dynamic conditions using flow chambers. To this end, mucus or mucins can be immobilized in wells on microtiter plates [120,121], and bacterial binding can be quantified through a variety of methods, including quantitative qPCR [122], enzyme-linked immunosorbent assay [122], or fluorometric quantification of fluorescently labelled bacteria [123]. The use of flow chambers in such a system allows for shear force to be controlled, thereby simulating the microbe–mucus interactions under dynamic conditions [124].

The short-term culture of organ pieces from animal models or human patients was established as an in vitro organ culture (IVOC) model [125], which was later improved through the polarized IVOC (pIVOC) model in which the tissue is fixed by a Snapwell support in a Transwell to limit bacterial contact to the mucosal side of the tissue [126]. A study applying the pIVOC system using duodenal explants identified the localization of *L. reuteri* in the mucus layer without penetrating the epithelium [127]. Subsequent incubation with EPEC, previously shown to induce interleukin-8 expression [128], in this system showed a reduction in EPEC adhesion mediated by *L. reuteri* [127]. Succeeding the pIVOC system, Gustafsson et al. established a short-term ex vivo intestinal explant culture model using horizontal perfusion chambers, which allowed studying mucus formation, properties, and thickness [8]. Subsequent studies have investigated mucus penetrability and goblet cell responses to microbial stimuli in murine models and patient biopsies, demonstrating that structural weakening and bacterial penetration of the mucus layer are early events in UC and that the normalization of the mucus layers requires long-term microbial colonization [24,73,77,82]. Around the same time, Sato et al. developed a new and now widely applied technology of intestinal organoid cultures which represent an ex vivo model for the long-term culture of self-propagating three-dimensional spheres of primary epithelial crypts or stem cells [129,130]. While the clear advantage of this model is its self-propagating nature and long-term expansion, the luminal side of the intestinal epithelial cells is confined to the inside of the growing spheres, meaning that bacterial association or infection experiments rely on microinjection into the sphere. The not so trivial execution of microinjection in a culture well containing up to hundreds of organoids leads to the development of two-dimensional systems with primary intestinal cells grown as monolayers that form a functional mucus barrier, which can be further increased through the basolateral addition of primary macrophages [131,132,133].

While the use of in vitro and ex vivo model systems increases our understanding of central mechanisms involved in microbe–mucus interactions by investigating the role of specific bacteria and bacterial products and selected mucins, in vivo animal models are indispensable to understand both the mechanisms and consequences of microbe–mucus interactions within the complex host. To date, several genetically engineered rodent models displaying alterations in mucins and mucin glycosylation have been generated. In addition, non-mucin related rodent models display mucus alterations and are often associated with inflammation and/or cancer (listed in Figure 2). Of these, the rodent models relevant to colon carcinogenesis will be discussed here.

As described in Section 5 of this review, the *Muc2*^−/−^ mice spontaneously develop tumors [80] and colitis [81] and display microbial dysbiosis [134,135]. As the major mucin of the intestinal mucus layer, studies using the *Muc2*^−/−^ mouse model have provided solid data rendering this mucin as a crucial component of a functional defense barrier in healthy and diseased subjects. Increased expression of underglycosylated MUC1 is seen in adenomatous polyps and colorectal adenocarcinomas [136,137] and leads to exposure of cryptic epitopes that can be recognized by cytotoxic T cells and trigger anti-MUC1 immune responses [138]. Interventions to target tumor-associated antigens such as MUC1 could be used in anti-tumor strategies. Transgenic *Muc1* (Tg*Muc1*) mice are useful for crossing with existing cancer model strains for MUC1-targeted tumor immunotherapy, as has been shown for Tg*Muc1* mice crossed with the Apc^MIN/+^ mouse model that forms spontaneous adenomatous polyps [139]. Colorectal cancer is associated with a loss of core-1, core-3, and core-4-derived *O*-glycans [92]. Indeed, mice that lack core-3-derived glycans display an accelerated colorectal tumorigenesis phenotype and increased susceptibility to dextran sodium sulfate (DSS)-induced colitis [140]. The double knockout of core-1 and core-3-derived *O*-glycans in mice results in spontaneous microbial dysbiosis-driven CAC [86]. Our own nATF6^IEC^ transgenic mouse model of ER stress (discussed in Section 5) presents a model displaying mucus barrier alterations and microbial dysbiosis preceding spontaneous colon tumorigenesis [105]. This model thus represents a mouse model of a non-mucin-related genetic alteration that clearly associates intestinal mucus and the microbiota with colorectal tumorigenesis, yet the alterations in mucus characteristics and microbe–mucus interactions involved in disease pathogenesis are still not fully understood. In addition to rodent models, mucus physiology is also studied in non-rodent models such as pigs and zebrafish [141,142]. Together, the above model systems that range from in vitro to in vivo methodologies provide a repertoire of approaches to successfully investigate microbe–mucus interactions in simple and more complex systems and study potential novel therapeutic tactics in CRC.

## 7. Conclusions and Treatment Potential

With the intestinal mucus layer presenting the first and central interface between the host and microbes, it provides a promising target that has only recently come into focus. While our understanding of interactions between microbes and intestinal mucus is increasing, it remains incomplete. Furthermore, the complex molecular nature of mucus remains poorly understood and requires additional investigation. Probiotics constitute live beneficial bacteria that confer health benefits to the host. Examples of mucus barrier-promoting probiotics include *Bifidobacterium longum* and *Lactobacillus reuteri* [143,144]. Evidently, bacterial products alone are sufficient to strengthen the mucus layer [64]. In light of the bidirectional interaction of microbes and mucus, targeting the gut microbiota to increase the mucosal barrier forms a major research goal. Furthermore, studies such as the recent work by Bergstrom et al. investigating fecal-associated mucus [28] provide new insights into microbiota metabolism and composition and may lead to noninvasive strategies such as fecal mucus screening for disease diagnosis. Experimental efforts need to address not only how individual bacteria interact with and utilize specific mucin glycans, but also how this affects mucus expression, glycosylation, and secretion. An obvious limitation of many studies is the inability to reflect neither the complex nature of the host mucin–glycan repertoire nor the interplay between different members of the entire intestinal microbial community, which both have a profound effect on microbe–mucus interactions and the consequence of the latter. Nevertheless, better understanding of microbe–mucus interactions and their effect on the mucus barrier is essential for the rational design of novel therapeutic agents to enhance the protective capacity of colonic mucus as a treatment approach for intestinal pathologies, including IBD and CRC.
